# “There’s no better medicine than my outdoors”: understanding the importance of physical activity in rural and remote First Nations communities in Northern British Columbia, Canada

**DOI:** 10.1186/s12966-026-01892-2

**Published:** 2026-03-21

**Authors:** Sunaina Chopra, Travis Holyk, Stacy Maddocks, Suzanne Huot, Pat G. Camp

**Affiliations:** 1 Graduate Programs in Rehabilitation Sciences, University of British Columbia, Vancouver, BC Canada; 2 Centre for Heart Lung Innovation, University of British Columbia, Vancouver, BC Canada; 3 Carrier Sekani Family Services, Prince George, BC Canada; 4 Palmerston North Regional Hospital, 50 Ruahine Street, Roslyn, Palmerston North, 4442 New Zealand; 5 University of KwaZulu-Natal Westville, Durban, 3629 South Africa; 6 Department of Occupational Science & Occupational Therapy, University of British Columbia, Vancouver, BC Canada; 7 Department of Physical Therapy, University of British Columbia, Vancouver, BC Canada

**Keywords:** Community-based participatory research, First Nations, Photovoice, Physical activity, Qualitative

## Abstract

**Background:**

While Canadian First Nations communities actively participate in sport, cultural, and land-based activities, they face various barriers to physical activity. Despite calls for increased support at all government levels, access to suitable programs for those with chronic conditions in particular remains limited. As part of an ongoing partnership between the University of British Columbia and Carrier Sekani Family Services (CSFS), this qualitative study aimed to explore current values, perceived barriers, and potential facilitators of physical activity among people living with chronic health conditions in rural and remote First Nations communities in northern British Columbia.

**Methods:**

This qualitative study is part of a larger mixed-methods, community-based participatory action project with CSFS titled *Niwh Yizt’iyh Hilht’iz Nets’eelh’iyh* – “Strengthening our Bodies”. Semi-structured interviews, with optional Photovoice, were conducted with participants aged 12 years and older who had at least one chronic condition and belonged to First Nations communities served by CSFS. Participants were recruited from a prior community-based physical activity survey study that captured brief information on current physical activity experiences in their community. Data were analyzed using reflexive thematic analysis, guided by Indigenous Wholistic Theory (a framework emphasizing the interconnected and cyclical physical, mental, emotional, and spiritual dimensions of health and wellness) to ensure cultural relevance. Coding progressed from semantic to latent themes, supported by NVivo and reflexive journaling.

**Results:**

Interviews were conducted with 29 participants from three communities (69% female, mean age: 55 years old), all managing various chronic conditions such as diabetes, arthritis, and cardiovascular disease. The identified themes were: 1) Integrating cultural values with physical activity to manage chronic disease, 2) Cultural disconnection: A barrier to health and physical activity, and 3) Community approaches to facilitate physical activity in chronic disease. Together, these themes highlight the role of culture, supportive community structures, and tailored approaches in physical activity engagement.

**Conclusion:**

To strengthen physical activity programming, service providers should ensure initiatives are aligned with community values, address identified barriers, and encourage locally recognized facilitators. These insights will empower health care organizations and communities to develop targeted, inclusive strategies for physical activity programming tailored to individuals with chronic conditions.

## Background

For generations, Indigenous people across the globe have engaged in outdoor physical activity on their ancestral lands as a source of healing and connection. The values, languages, and cultures of many Indigenous people are rooted in their engagement with the land around them [1]. The land prompts memories, stories, and traditional ways of life [2]. On this land, there are opportunities to engage in various cultural and physical activities, such as berry picking, hunting, and fishing. Beyond the benefits these outdoor land-based activities have on the community and local environment [3], they also offer a myriad of health and wellness opportunities to individual Indigenous people.

Outdoor exercise across all seasons has the potential to reduce the negative impact of chronic health conditions; a promising avenue of pursuit used in other contexts [4]. For instance, many people find joy and motivation in outdoor activities year-round, including in cold weather and snowy conditions [5]. Self-esteem and mood improve in participants who take part in outdoor activities, often referred to as “green exercise” [6]. Breast cancer survivors and individuals experiencing mental health challenges have described exercise in nature as a source of healing and emotional regulation [6, 7]. These findings highlight the benefits of outdoor engagement and suggest the usefulness of land-based activities for Indigenous people managing similar chronic conditions.

Indigenous people face disproportionately high rates of chronic conditions due to the ongoing impacts of colonialism and assimilation [8]. In Canada, several First Nations initiatives exist that focus on outdoor activity as one way to improve wellness and reduce the impact of chronic disease. For example, a walking program in a First Nation community in Lytton, British Columbia (BC) demonstrated measurable improvements in cardiovascular health [9], while land-based programming in the northern territories has been credited with enhancing self-esteem and reinforcing cultural identity [10]. Culturally grounded activities among First Nation community members living with diabetes have also shown promise in reducing stress and improving symptom management [11]. These examples highlight the relevance of integrating traditional cultural practices into chronic disease interventions.

Much of our previous knowledge of the perspectives and preferences of land-based activities in First Nations communities is from urban settings [12, 13]. Our understanding of preferred land-based activities in the context of rural and remote northern communities is limited. Members of these communities live in unique geographic contexts that shape their experiences and priorities [14]. In addition, existing research often focuses on programs for specific conditions, such as diabetes [11, 15], arthritis [16] and cardiovascular disease [9, 12], but communities in remote and rural areas may not be able to sustain a single-disease-specific approach. Multi-morbidity is common, and there is a call to action to improve services for all conditions [13]. Incorporating the perspectives and preferences of First Nations participants with chronic conditions living in rural and remote communities is essential for developing initiatives that align with their goals, supporting both long-term adherence and cultural relevance for Indigenous people [17, 18]. However, to ensure the relevance and effectiveness of programming in rural and remote Indigenous communities, it is important to prioritize the voices of individuals across varied ages, genders, and health conditions to create programs that will enable inclusive participation.

The aim of this qualitative study was to explore the lived experiences of physical activity among individuals living with chronic conditions in rural and remote First Nations communities in northern British Columbia, in order to inform community-based and culturally responsive physical activity programs.

## Methods

### Study design

This qualitative study used a community-based, participatory approach involving semi-structured interviews and a Photovoice project with members of First Nations communities living in northern British Columbia.

### Study context

Guided by the phrase “Creating Wellness Together,” Carrier Sekani Family Services (CSFS) delivers programs and supports that promote the health and wellness of Nations across the Carrier and Sekani territories in British Columbia (BC). Traditionally spanning more than 76,000 km^2^ in north-central BC, these territories are now home to over 10,000 Carrier and Sekani First Nations people. CSFS was formed to restore self-determination for Carrier and Sekani First Nations in the areas of health, social, family, and legal services. Within its health department, CSFS adopts a holistic approach to strengthen wellness at the individual, family, community, and Nation levels. Its primary care model includes services such as nursing, mental health, substance use and addictions support, and allied health care. In response to community feedback gathered through a health evaluation, CSFS has been expanding initiatives in the Health Promotion and Physical Literacy department. Working alongside physiotherapists, occupational therapists, coaches, and other fitness professionals, the department is focused on developing sustainable, community-driven programs.

In 2015, researchers at the University of British Columbia (UBC) connected with CSFS to initiate meaningful community engagement through lung health events, assessing community needs, and pursuing funding for research initiatives aligned with the communities' priorities. This joint community-based participatory research initiative between UBC and CSFS became known as *Niwh Yizt’iyh Hilht’iz Nets’eelh’iyh*, which means “Strengthening our Bodies” in traditional Dakelh language. This collaborative process highlighted a critical need for physical activity programs tailored to all individuals in the community, including those at-risk and experiencing chronic conditions. This need was reaffirmed during the CSFS Health Evaluation in 2022, where calls to enhance physical activity programming were reiterated.

Since then, our project team has continued to reflect this focus on community-centered engagement, bringing together members from diverse academic disciplines and professional roles. Our social identities intersect across our commitments to improving health and wellness outcomes and are shaped by varied racial, social, ethnic, cultural, and socioeconomic backgrounds and positionalities. These intersecting identities, alongside our collective personal and professional experiences with First Nations communities, allow us to approach this work from multiple perspectives. In embracing this diversity, we conducted research that was academically rigorous, and deeply respectful and responsive to the communities.

### Recruitment

Recruitment for this study was conducted following initial engagement at local health fairs within the community, where information about the project was shared. These events generated interest and support from community leadership, which facilitated recruitment in three First Nations communities served by CSFS (two rural and one remote). Eligibility criteria, including the age range, were determined in collaboration with CSFS and community leadership to align with community priorities and goals. Eligibility criteria were the following: aged 12 years or older, completion of a physical activity survey in a previous phase of the larger project [19], consent for future contact, and self-reported experience of a chronic condition or symptoms limiting their physical activity. Recruitment was also supported through collaboration with CSFS staff and community leadership, accompanied by the distribution of flyers within communities and on social media platforms. To ensure representation across gender, age, and health conditions, purposeful sampling techniques were used. Participants who completed an interview received a $75 gift card honorarium.

Ethics approval was obtained from both the CSFS (approved October 2022) and the UBC Research Ethics Boards (#H18-01640, approved October 2018). In addition to following Western research ethics guidelines, this study adhered to the First Nations Information Governance Centre’s Principles of OCAP™, ensuring that the communities maintained authority over their data [20]. All researchers involved completed the CSFS *Nowh Guna’* Carrier Cultural Training. Written informed consent was obtained from all participants, who were also reminded of their right to withdraw consent at any stage of the research process. Community members were informed that their participation was completely voluntary, and confidentiality was assured. To ensure this, participant identities were protected using coding systems and pseudonyms. Both physical and digital copies of the data were securely stored, with access restricted to the research team per UBC’s Privacy and Information Security guidelines.

### Data collection

Demographic information, including participants’ self-reported age, gender, community affiliation/association, and chronic health conditions, was derived from survey data collected during the previous phase of the study. The semi-structured interviews were conducted in English in-person at a local community building (e.g., health center, Band office) or over Zoom by the first author. Before each interview, participants were provided with a detailed overview of the interview process, including the nature of the questions, anticipated topics for exploration and the estimated duration. Participants were provided information about the Photovoice project and the option to submit photos related to their physical activity experience. If participants were interested in the Photovoice project, their photos were sent to the interviewer. Each in-person interview was audio recorded using a digital voice recorder. Similarly, over Zoom, a review of the interview process and verbal agreement were given before the recording commenced. All data were uploaded to an encrypted digital folder. The final sample size was determined based on practical considerations (including project and funding timelines for the need to advance to subsequent phases), and the desired level of data saturation. Saturation was assessed iteratively during both the data collection and analytical process, and was considered achieved when subsequent interviews no longer contributed meaningful new insights (e.g., contexts, perspectives, depth) to the development of themes.

#### Interview questions

Drawing from the initial survey of this project and question frameworks from previous studies in this area [21, 22], interview questions were developed in collaboration with CSFS to ensure they were culturally appropriate and relevant to their member communities. While the questions were not formally piloted, the research team reflected on their clarity and applicability after each set of interviews to determine if revisions were needed. Minor revisions were made to simplify language by reducing jargon and adding prompts to encourage more open-ended, in-depth responses. Refinements were informed by reviews of transcripts from early interviews, community context, and findings from the prior community-based physical activity survey. Consistency in the core topics explored across participants was maintained. The questions comprised a total of 10 topics summarized in Table 1.

**Table 1:** List of topics in the interview guide

1	Types of physical activities participants engage in and the locations where these activities occur
2	Forms of physical activity commonly undertaken by participants
3	Participant suggestions for promoting walking within the community
4	Identification of potential sites for developing walking paths in the community
5	Community-level barriers that hinder participation in physical activity
6	Health-related and psychosocial factors that limit engagement in physical activity
7	Facilitators and supports that enable or encourage physical activity within the community
8	Perceived changes in physical activity patterns over time
9	Strategies for integrating cultural practices into future physical activity programming
10	Attributes and competencies considered important in an effective physical activity instructor

#### Photovoice methodology

Incorporating a Photovoice project with interviews is a culturally appropriate method that aligns with how Indigenous knowledge is traditionally shared [23]. Using guidelines from previous projects conducted, participants had the option to complete a Photovoice project and voluntarily submit photos they took using their devices. We encouraged photos related to activities participants enjoy doing, locations they are active in, barriers they experience to being active, and facilitators to their activity level. If a participant shared their photos, a tailored question guide was used during the interview sessions. This guide enabled participants to reflect on the photos they provided, encouraging them to share insights and experiences through their chosen photos. Using these photos as visual aids, participants elaborated on their stories, which fostered a mutual, in-depth understanding of their current physical activity experience in a geographical area that many of the researchers are not from. Photo release consent was obtained from participants who submitted photos of themselves or with other people in them.

### Data analysis

The analysis was guided by Indigenous Wholistic Theory, as described by Anishinaabe scholar Kathy Absolon [24]. This theory was applied to explore the physical, mental, emotional, and spiritual impacts of physical activity, as well as its influence on participants’ families, communities, and local environments. Grounded in ancestral worldviews and a four-directional understanding, Wholistic Theory emphasizes relationality and interconnectedness, providing a culturally relevant framework to generate recommendations toward future physical activity programming that are empowering and responsive to community needs.

**Fig. 1:**
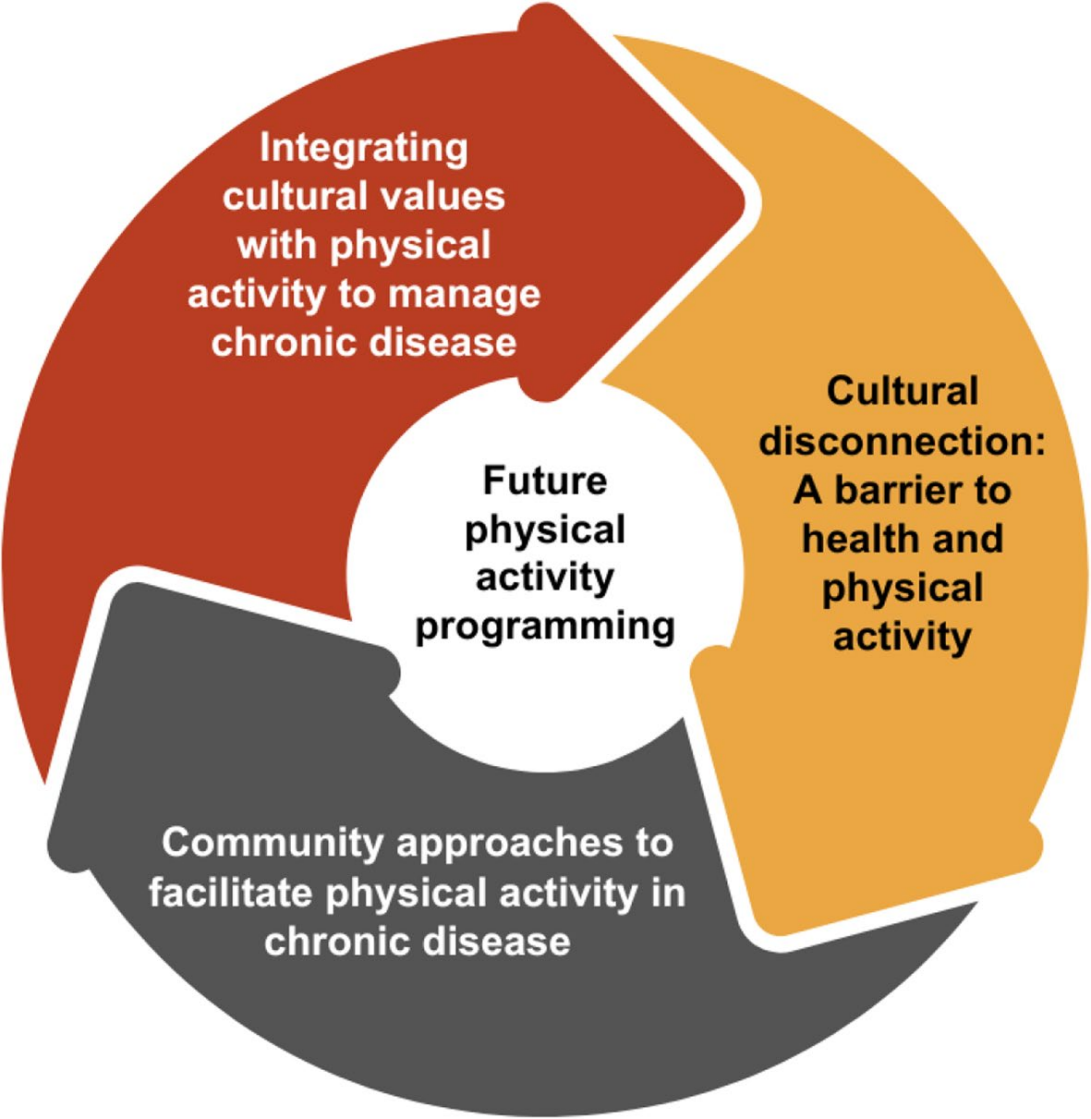
Summary of themes constructed during analysis

Descriptive statistics were calculated for participant characteristics using the survey data. Interviews were transcribed verbatim and deidentified. A semi-naturalism approach was applied to include only cues that added meaningful context while minimizing the risk of misinterpretation [25–27]. Participants who provided contact information were given their transcripts with a 10-day window for accuracy review. Transcripts were re-read to identify initial patterns before uploading transcripts and photos into NVivo for coding and analysis. Reflexive thematic analysis [27] was used by the first author, following an inductive process from semantic to latent codes, which were then grouped into sub-themes and refined into themes iteratively. The first author approached the data with a contextualist epistemology and critical realist ontology [27], which focused on the development of experiential themes that referred to participants’ viewpoints, intentions, beliefs, and experiences as they were evident in the data. Interpretations and themes were further reviewed through discussions with the third author, providing an additional layer of reflexivity and verification. Theme names and descriptions were then confirmed against the data, research questions, and existing literature, with an overview figure created using NVivo R1 (Lumivero, LLC) and Canva (Fig. 1). Reflexive journaling was maintained throughout to enhance credibility, while participant characteristics and contextual details were included with quotes to support transferability [27].

## Results

Twenty-nine individuals consented to participate in the study. The demographic characteristics of the participants can be seen in Table 2. This group of participants had a mean age of 55 years (range: 20–84 years old) and were mostly female (69%). The average interview length was 39 min (SD = 17, range: 17–89 min). Most interviews (n = 27, 93%) were conducted in-person in the community’s health center or band office.

**Table 2:** Demographic characteristics of participants

**Number of participants**	29
**Age (years)**	
Mean ± SD	55 ± 16
Range	20—84
**Gender, n (%)**	
Female	20 (69%)
Male	9 (31%)
**Self-reported health conditions**	Arthritis Cancer Chronic fatigue Chronic kidney disease Chronic pain Heart conditions (e.g., previous heart attacks) Respiratory conditions (e.g., COPD) Mental health Stroke

Three themes were constructed during the analysis (Fig. 1). The themes were: 1) Integrating cultural values with physical activity to manage chronic disease, 2) Cultural disconnection: a barrier to health and physical activity, and 3) Community approaches to facilitate physical activity in chronic disease (Table 3).

**Table 3:** Summary of themes and sub-themes from the interview and Photovoice data

Theme	Sub-theme	Codes
Integrating cultural values with physical activity to manage chronic disease	Appreciation for traditional activities	Engagement in seasonal, land-based activities
		Enjoyment in cultural and group activities
		Intergenerational continuation of cultural practices
	Importance of kinship	Physical activity as an opportunity for connection and cohesion
		Growing concern for the lack of physical activity among youth and the influence of technology
		Significance of Elders for engagement
	Physical activity provides an opportunity for improving health and wellness	Assigning health benefits to a personal connection with nature and culture
		The healing ability of the land
		Improved cultural identity through traditional practices
		The land as a source of nourishment
		The advantages of rural locations
Cultural disconnection: A barrier to health and physical activity	The ongoing effects of colonialism	Lack of appropriate community resources and equipment
		Trauma and racism
		Competing occupational demands impair opportunities for recreational physical activity
	A changing, unpredictable environment	Unsupervised pets and wild animals
		Deforestation
		Wildfires
		Loss of cultural practices has impacted environmental health
		Quality of aquatic resources
		Climate instability
	Influence of COVID-19 and other chronic conditions	Physical health
		Mental health
Community approaches to facilitate physical activity in chronic disease	Investment is needed to expand infrastructure, technology, and financial support	Infrastructure
		Wearable monitoring devices
		Increasing resources through financial support
	Culturally-focused programming	Passing down of ancestral knowledge and practices
		Culturally competent, appropriately skilled instructors
		The need to incorporate language
	Tailored physical activity, group programs	Gender, especially for women
		Age, especially for youth and Elders
		Family, especially young and new parents

### Integrating cultural values with physical activity to manage chronic disease

**Fig. 2:**
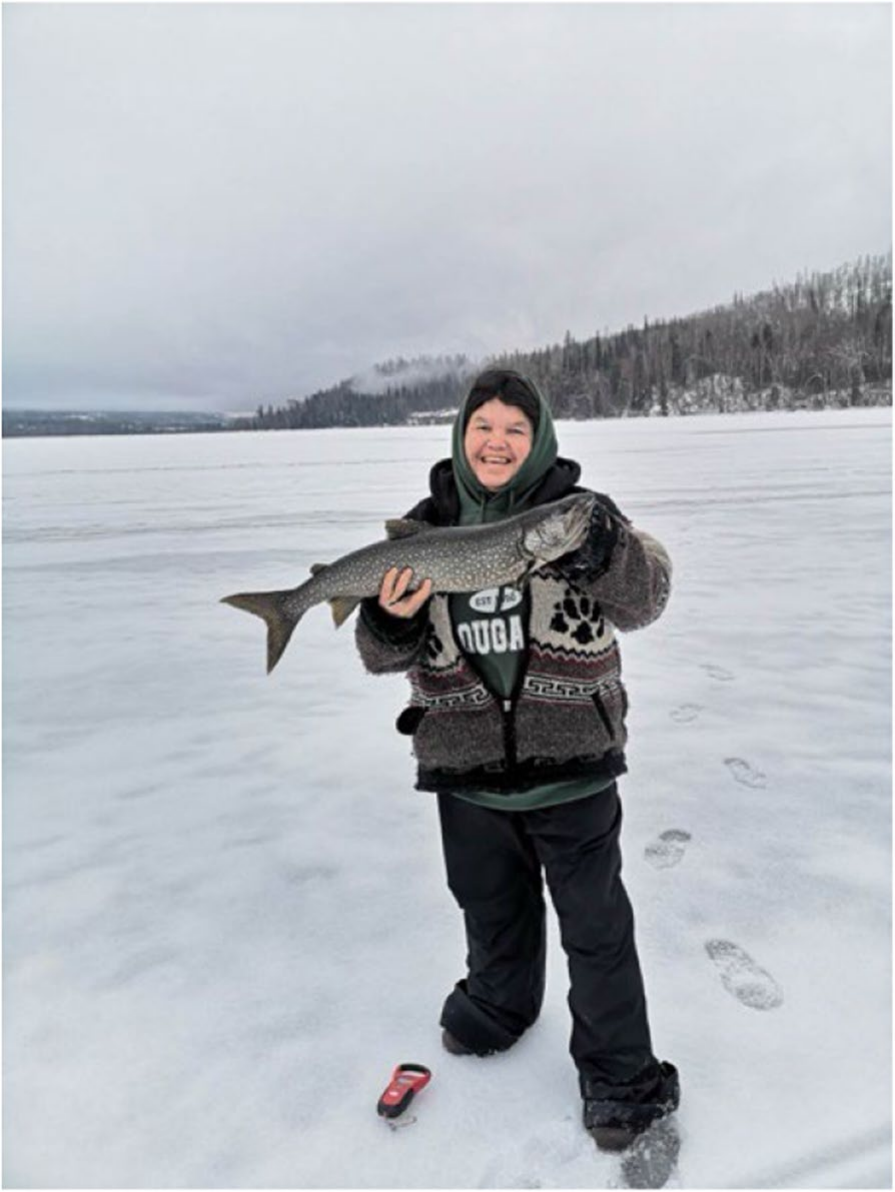
Photovoice project submissions that relate to how physical activity aligns with cultural values. Figure 2 depicts a woman happily holding a fish she caught while ice fishing, one of her favorite winter activities. [Permission for use of photos was obtained by the participants through written consent, approved by UBC and CSFS.]

**Fig. 3:**
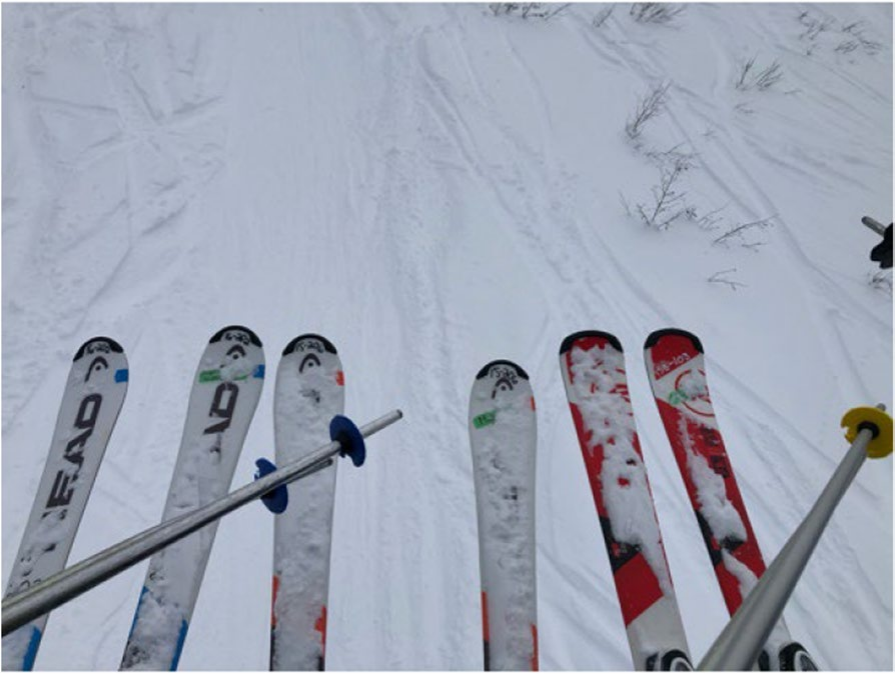
Photovoice project submissions that relate to how physical activity aligns with cultural values. Figure 3 depicts a woman with her two children on a ski lift going skiing at a nearby mountain

**Fig. 4:**
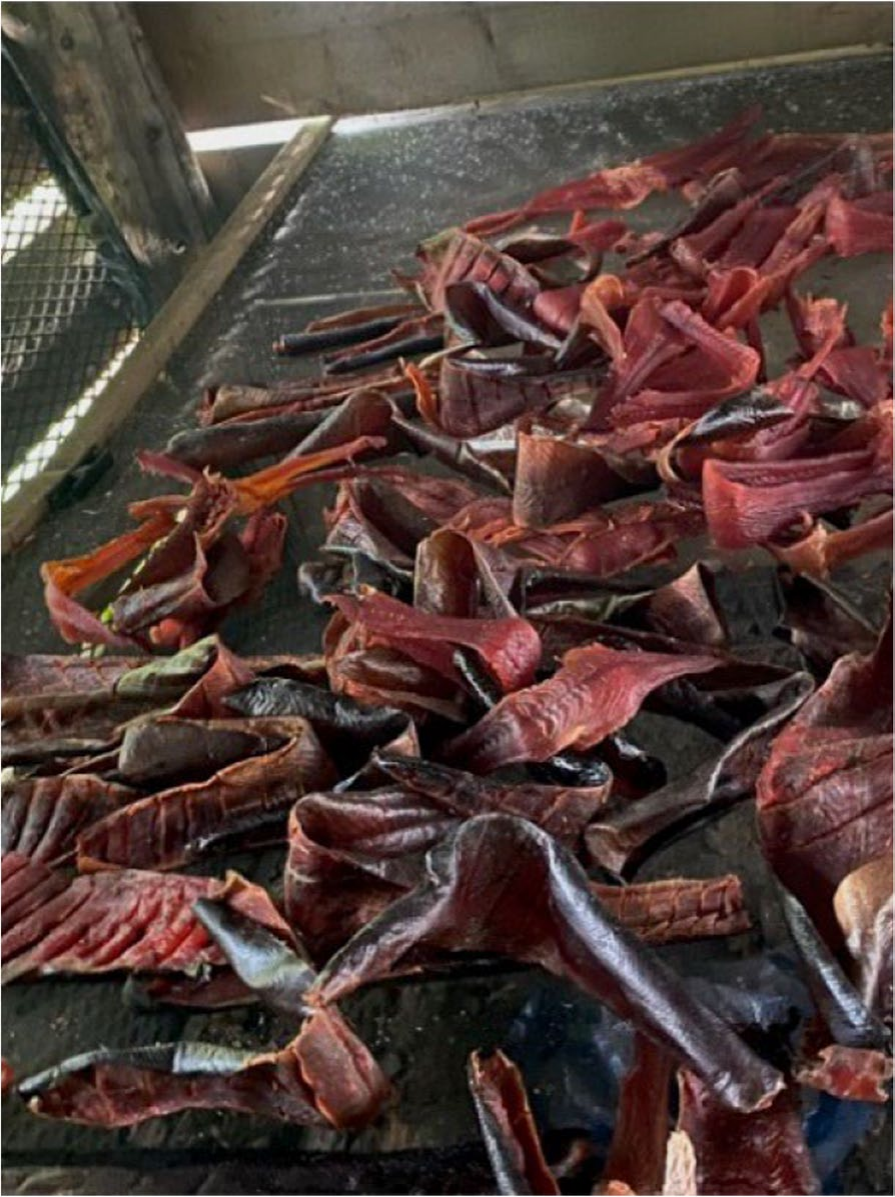
Photovoice project submissions that relate to how physical activity aligns with cultural values. Figure 4 depicts harvested salmon being dried on a rack for later storage and preservation

Participants described how the current physical activity experience in the rural and remote areas of north-central BC aligns with cultural values practiced by many Carrier and Sekani people (Fig. 2, 3 and 4). For example, they frequently highlighted their enjoyment of outdoor activities on their ancestral lands with family members:

“*I love going out on the land and berry picking and medicine gathering and just trying to gain all the knowledge that my mom has. It’s interesting and I love doing it. I try to teach as much of that to the two kids and not only them, there’s my other niece that my sister has, and she usually comes with us, and then my niece and nephew that come up from Prince George, they usually tag along, and we try to teach them as well*.”—54-year-old female participant describing what activities she enjoys doing with her family.

In addition to land-based practices, many participants enjoyed various cultural activities, such as the *Lahal* – “Bone Stick” game, dancing, and drumming. *Lahal* was described as a guessing game that involves two teams seated across from one another, where game pieces are hidden in the players’ hands, traditionally made from small animal bones that were carved or marked in a culturally specific way. Many participants enjoyed the positive group atmosphere that these types of activities created. However, participants noted that physical activity traditions are changing, especially in youth. They commented on how modern advances, such as cell phones, video games, and social media, have led to a decline in youth activity and that technology is keeping youth inside and not as active as their parents, grandparents, and ancestors were:

*“When I was a kid, we used to walk everywhere. We used to walk across to that mountain. I’m not seeing that there’s too much walking. Some of them, they do walk, but not a whole lot.”*—80-year-old female discussing the current lack of activity among youth compared to the past.

“*And now these young kids, not one of them today, they put on snowshoes, not one. And I don’t think some of them go out in the bush*.”—78-year-old male participant describing the difference in activity among youth compared to when he was younger.

Similarly, many participants also reported a decline in the activity levels of their community Elders. This was a concern for many participants because they emphasized how important Elders are in passing down traditional knowledge and community cohesion.

### Cultural disconnection: a barrier to health and physical activity

**Fig. 5:**
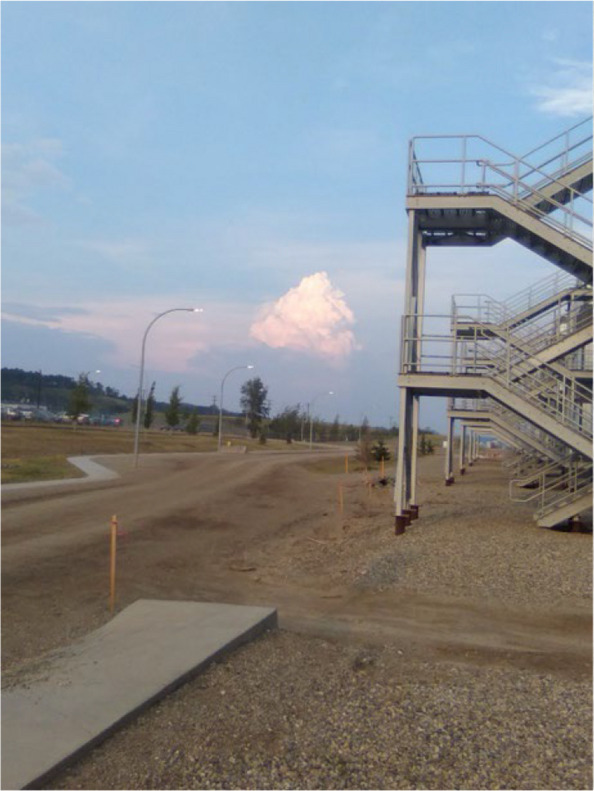
Photovoice project submissions on restoring historical ways to reduce barriers to physical activity. Figure 5 represents a participant’s workplace, which they cited as their primary location and source of physical activity

**Fig. 6:**
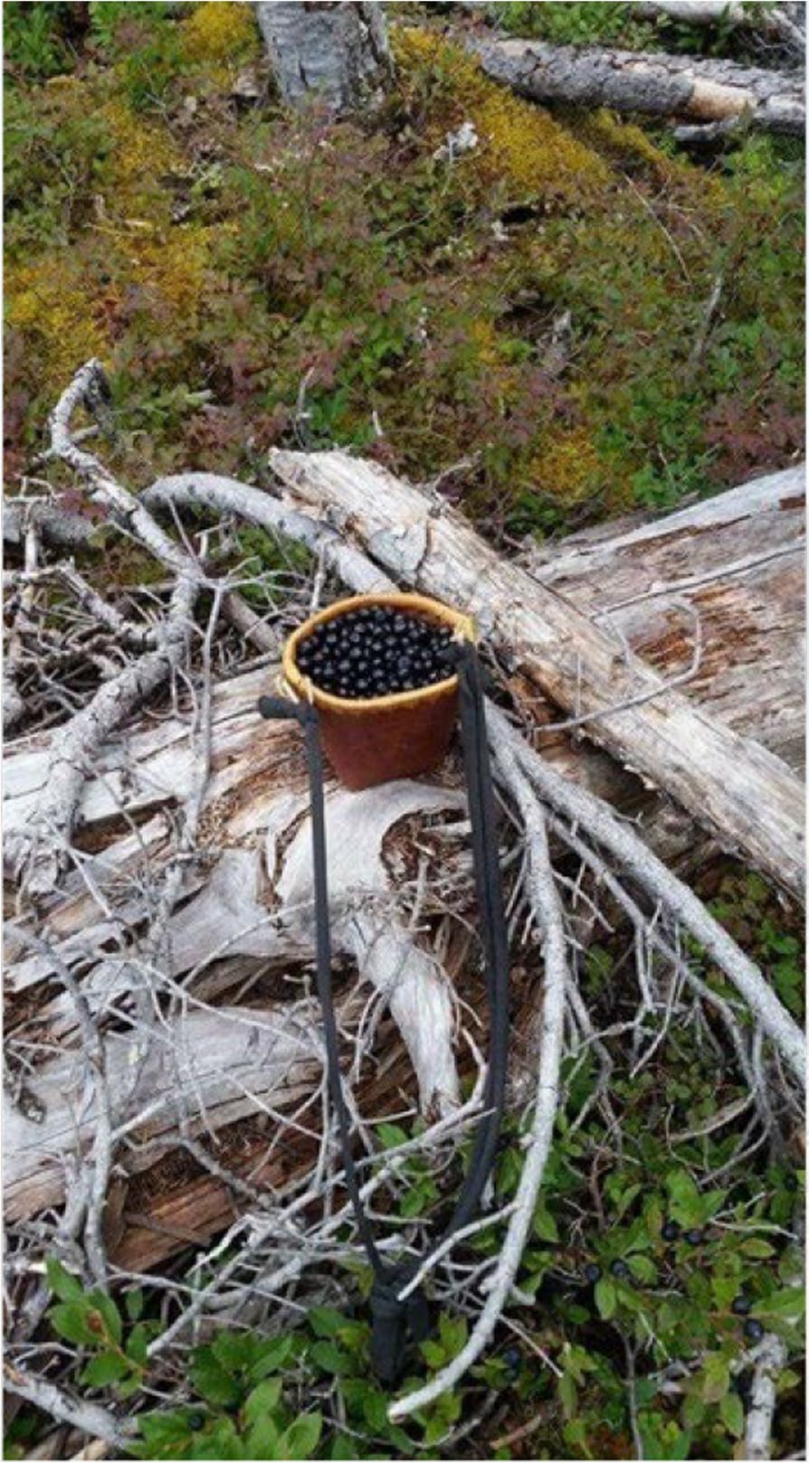
Photovoice project submissions on restoring historical ways to reduce barriers to physical activity. Figure 6 depicts a berry-picking area that one participant would often visit and encounter bears in

**Fig. 7:**
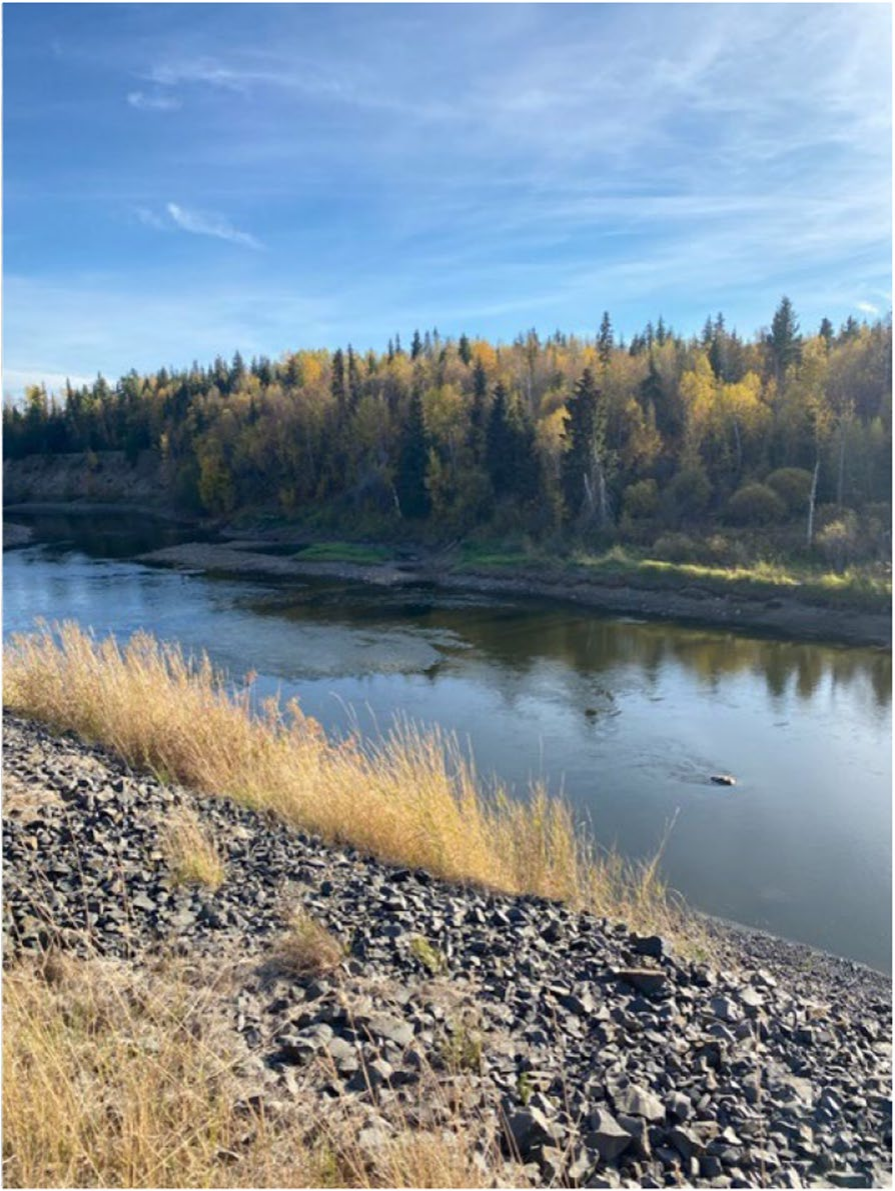
Photovoice project submissions on restoring historical ways to reduce barriers to physical activity. Figure 7 is a river located close to one community where salmon are known to often swim, but have recently declined in numbers

To reduce this decline in activity, many participants stated there is a need for programming focused on reconnecting the community to their land (Fig. 5, 6 and 7). These types of activities provided many benefits to the participants, including cultural connection, healing ability, strengthening cultural identity, and providing nourishment:

“A lot of the way we were taught, we were taught to live off the lands. You have to harvest everything. I like doing that. I’d rather do that harvesting than having to go into a grocery store and buy food. You actually done it yourself instead of having to go buy whatever you need in a grocery store.”- 52-year-old female participant discussing the importance of harvesting food from the land, versus the convenience of buying food from a store.

Participants did report that this has been on a decline, though, due to wildfires and other climatic changes, such as drought, deforestation, and changes in local waterways. While participants felt that living in this environment was advantageous, they also emphasized the importance of passing on traditional knowledge and teachings to ensure children and youth are well-educated when engaging in outdoor activity due to the presence of wild, unsupervised animals:

*“Animals tend to go down to the river a lot in the summertime to get their fish and whatnot. And there was a lot of bears in the community from all the fires, but that again is kind of hard to control cause it’s just nature. But I think being more prepared for those kind of interactions with animals, like being bear aware and having the necessities for being in that situation.”*—30-year-old female participant describing environmental barriers experienced when being active outdoors.

Other participants did find that their rural setting was restricting their activity level, due to the lack of resources, for example. In the absence of these resources, most participants attribute their activity to their tasks at work, household chores, and family care. These participants believe that this diminished activity resulted in a loss of culture and connection among many community members. All participants asked for programming focused on restoring tradition and culture to reduce the risk of many chronic conditions that the participants experienced themselves:

*“When I would walk, I’d try to do a minimum of 15 min. But sometimes with COPD, you don’t feel like it, and I don’t like that feeling. I don’t feel good.”*—66-year-old male participant discussing why he may not be active and go for walks.

### Community approaches to facilitate physical activity in chronic disease

**Fig. 8:**
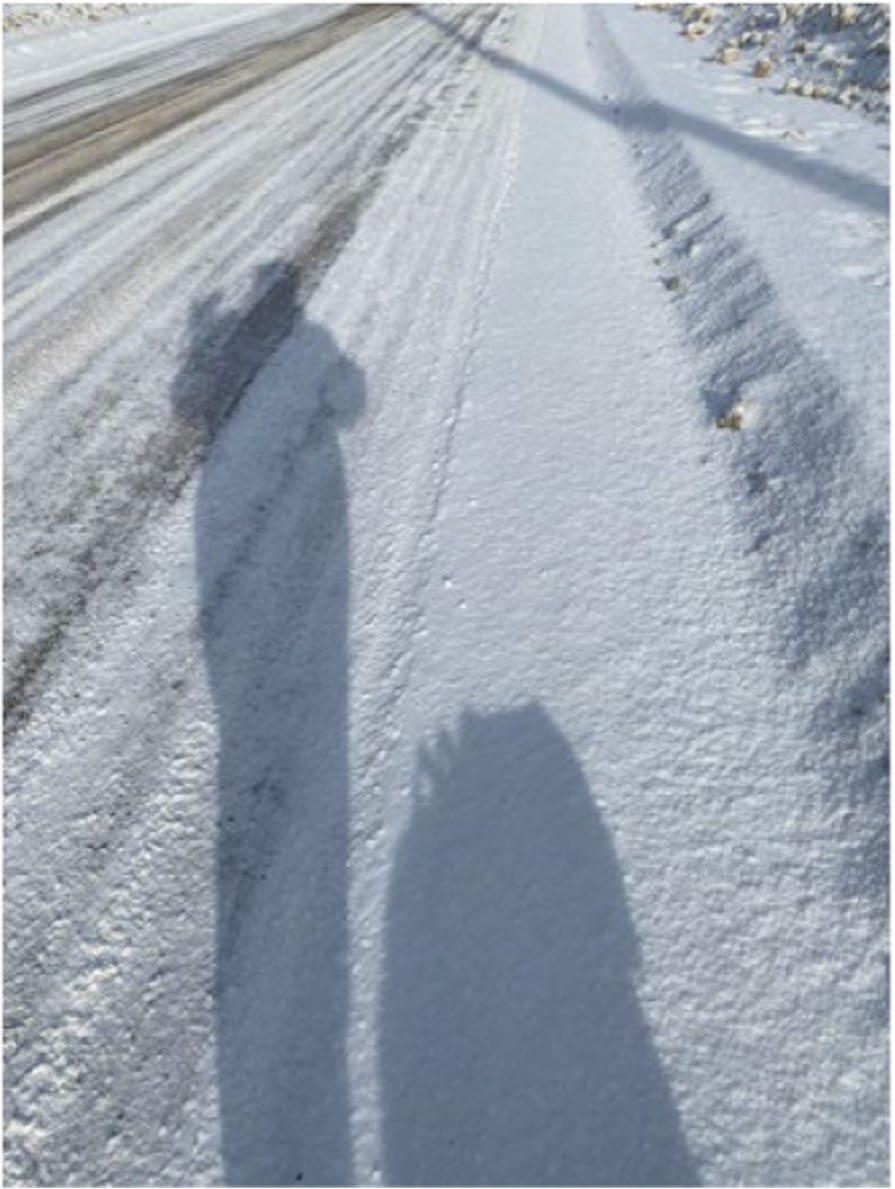
Photovoice project submissions that relate to how community-specific changes can facilitate physical activity. Figure 8 shows a participant walking on icy road conditions with her dog, and an example of why improvements to outdoor walking tracks or paths are needed

**Fig. 9:**
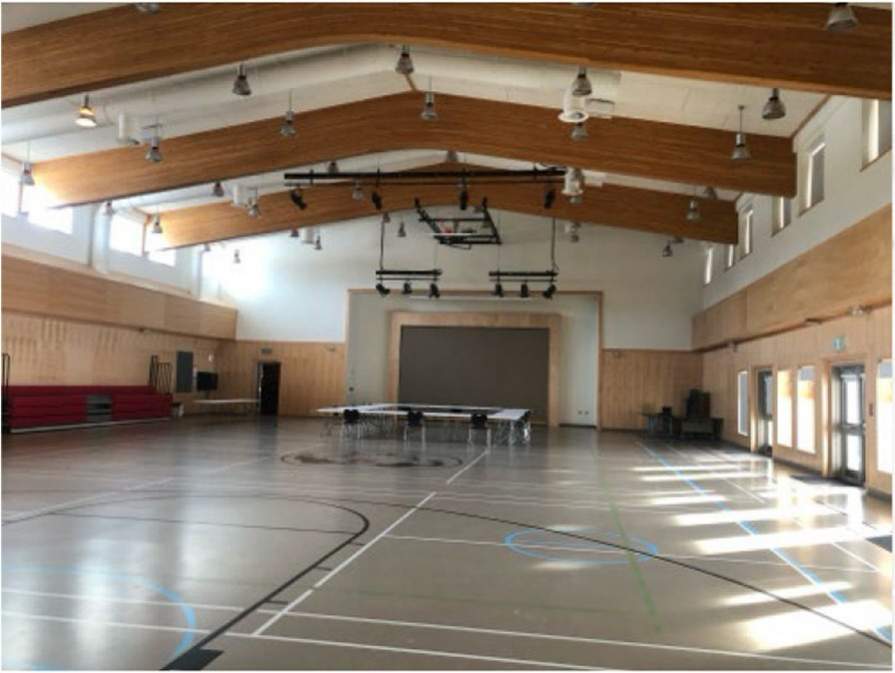
Photovoice project submissions that relate to how community-specific changes can facilitate physical activity. Figure 9 is the indoor gymnasium in one community that represents a space where group activities can be held

**Fig. 10:**
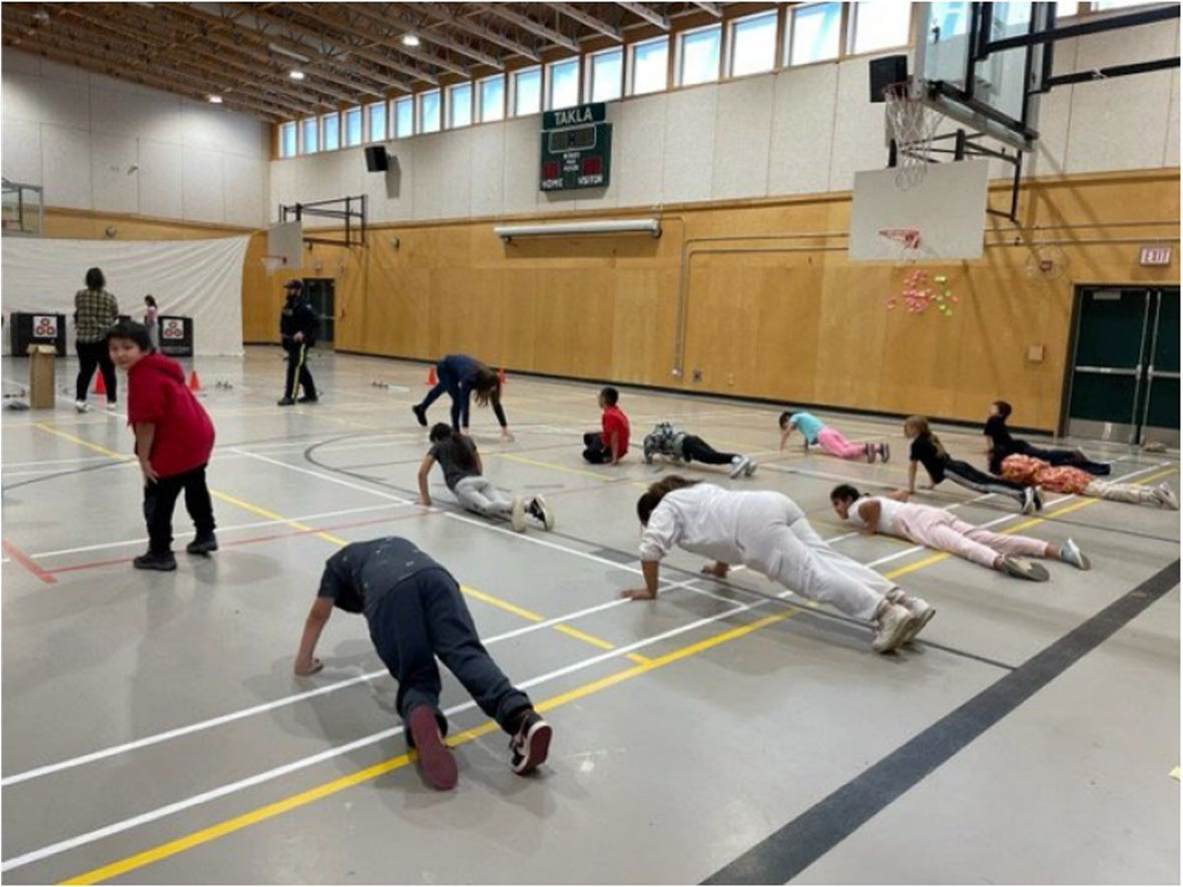
Photovoice project submissions that relate to how community-specific changes can facilitate physical activity. Figure 10 shows youth being active in a gymnasium in another community, which was one age group the participant requested there be a focus on

One strategy to prevent and manage chronic conditions for the entire community is to make changes to existing physical activity programming (Fig. 8, 9 and 10). Participants in three communities reported what specific changes needed to be implemented specifically for their community. In general, each community has different recommendations on types of infrastructure, group programming, and instructors.

While one community has large changes to infrastructure occurring, with many participants discussing the incoming ice arena:

*“I’m so excited [for the new arena] because I never skated since I was a kid. And to put on a pair of skates, I’d do it again. I know we have an arena and public skating in Fraser Lake, but that’s not as the same as being at home.”*—48-year-old female discussing the new arena coming to their community.

Other communities are looking for smaller changes, such as those expressing a need for a dedicated track or path, situated away from the highway to ensure safety:

*“There’s a North Shore Road that is a part of the loop that I do, but it’s a main road where people drive that don’t live in our community. They drive through our community to get to their homes. So it’s a really busy road. It’s always busy. So one single walking path would be sufficient and would be helpful. Just for safety reasons. People seem to speed through there.”*—61-year-old female describing the need for a walking path in the community for safety reasons.

This infrastructure would provide the opportunity to organize walking groups, which was of interest to the participants. The participant noted that such a pathway should be regularly maintained, including plowing and de-icing and equipped with anti-shock padding to accommodate individuals with arthritis and mobility challenges. They felt that building this track would significantly contribute to meeting their daily fitness goals, such as those associated with wearable technology like Fitbits.

In addition to this new infrastructure, there was a suggestion to facilitate more group programming that was gender-focused, such as a women’s group, or age-focused, such as a youth or Elders group. Regardless of the dynamic, many participants highlighted that these group programs have the potential to keep them accountable and motivated to attend regularly.

Another way participants would feel motivated to be active is the presence of instructors or teachers:

*“What makes a good teacher? A good listener, watching body language, knowing when something’s wrong with someone, supporting, just being there, not forcing kids to, or anyone, to do things that they shouldn’t do. Knowing their limitations and just role modelling, good role modelling.”*—55-year-old female with previous experience in early childhood education discussing what qualities make for a good instructor.

Participants reinforced that regardless of the activity or the participants involved, the instructor or teacher needs to prioritize the group’s needs. To participants, this meant not only educating them about wearable technology but also providing guidance on exercises beneficial for conditions like heart disease and offering dietary advice tailored to individuals with diabetes. The participant discussed these aspects as being important for instructors to build a sustainable, positive group atmosphere.

Finally, participants emphasized that language revitalization is a central goal within many communities and suggested that future physical activity programming should intentionally support this objective. A preference was expressed for family-based programming, as it provides opportunities to integrate language into the program context. Participants further indicated that community-led instruction would be essential, noting that instructors who are community members would bring both cultural competence and the skills necessary to enrich programming.

## Discussion

This study aimed to highlight experiences and recommendations around physical activity among many Carrier and Sekani First Nations in rural and remote north-central BC. While research has previously investigated this topic, this study stands out for the novel exploration of how land-based activity can support health and wellness across an array of chronic disease conditions in rural settings. The participants in our study have discussed how much they value activities that align with their cultural beliefs, and returning to regular engagement with these traditional practices could be greatly beneficial. Incorporating this feedback into how future programming should be designed and delivered holds promise to enhance activity levels in each community.

Many participants enjoyed engaging in land-based activities for the health and wellness benefits their traditional land offered [1]. The participants shared stories on the breadth of opportunities available for outdoor activity, such as berry picking, walking, and gathering traditional medicine. Across diverse activities, participants consistently emphasized the value and enjoyment of being physically active on their ancestral lands. This is consistent with existing research that highlights the profound impact these types of activities have on all the domains of health and wellness among Indigenous people [28–30]. However, beyond sustenance through hunting and gathering, these activities nourished the emotional and spiritual health of many participants. These land-based activities were deeply valued as integral to healing, cultural connection, and spiritual nourishment [10, 31].

The most common way participants were present and active on their land was simply through walking. Walking has been shown to improve symptoms in those with various conditions, including COPD and diabetes [32, 33], which is likely why many participants requested improvements to facilitate walking in their community. One request was to address concerns around safety due to difficult weather conditions and inadequate outdoor infrastructure, a common barrier in these northern settings [34, 35]. The construction of a walking path or track in the community could be a solution to this, which could contribute to improving walkability and encouraging this behavior [36].

However, other issues will not be so straightforward to fix. With the health and wellness of the community dependent on the local environment, initiatives must be taken to protect their lands, especially with the recent heightening of climate change. For example, many participants reported the greatest decline in their activity levels following wildfires. These have emerged as a growing and unpredictable threat in the summer, resulting in damage to traplines, scarcity of game populations, disappearance of berry bushes and medicinal plants, and more [37]. Even long-term environmental changes, such as the impacts of deforestation and changes in salmon populations in rivers, have caused a decline in outdoor activity in each community. Recognizing this relationship between health and land is vital to ensure the protection and preservation of these ancestral lands [1, 29].

This recognition of the interdependence between the land and well-being has the potential to benefit the entire community. For instance, as an easy, low-cost activity, outdoor walks may positively foster relationships with family, friends, and community members. Participants stressed the importance of involving as many people as possible in any activity, recognizing the role of group dynamics in initiating change and the potential for activity to influence multiple generations. This speaks to the importance of kinship and support networks in facilitating engagement [30, 38]. For example, many expressed interests in family programming to allow children, parents and grandparents to be active together. One advantage of this type of programming is that it has the potential to reduce concerns around wildlife, a worry many had when outdoors [35]. However, by having knowledgeable and equipped individuals present to protect the group, this can support land-based activity across all ages [35, 38]. These safe, group dynamics can be an excellent opportunity to encourage all community members to be active outdoors [34, 35, 39].

Building on that importance, participants noted there were two age cohorts that needed prioritizing: the young and the old. Focusing on the betterment of the younger generations was deemed important to many of the participants and aligned with findings that show how important land and cultural connections are for youth [40]. Participants viewed these efforts as a tool to prevent chronic conditions among children and youth, which could contribute to the development of a stronger, healthier future [8, 13]. However, participants equally discussed another key group on the other side of the age spectrum, and the biggest pillar of support in the community, their Elders. Participants insisted on activity programming and support for their Elders, such as walking groups, to ensure they can be healthy and happy for as long as possible [41].

Participants noted the importance of accommodating the barriers relating to chronic conditions. Each community had individuals with an array of symptoms across all ages who require accommodations in future programming. Without those modifications, this group will continue to be excluded from attending and participating in a program, a common issue reported in the literature [42, 43]. Ensuring that physical activity opportunities are accessible and inclusive is therefore essential for enabling equitable participation. The first step in resolving this would be to start small. Even little amounts of low-intensity activity can provide great benefits to individuals with chronic conditions, like diabetes [32]. By encouraging and integrating these brief bouts of activity throughout the day every day, those benefits can start to be observed in participants.

One tool that can help with that is wearable technology, a topic that was positively received by participants in this study. These devices can provide useful feedback and knowledge on physical activity, enabling wearers to make informed decisions about how they wish to pursue their unique daily goals [41, 44]. In rural and remote contexts, however, monitoring both physical activity and the environments in which it occurs presents unique challenges related to access and connectivity [45]. These considerations highlight the importance of implementing monitoring technologies in ways that are community-informed, culturally safe, and attentive to issues of equity, privacy, and Indigenous data governance [20, 45]. When thoughtfully applied, such tools may contribute not only to individual behavior change but also to evidence-informed improvements in community physical environments [45].

Another valuable way to center community engagement in physical activity is by honoring the joy and cultural significance embedded in these practices. Participants shared that laughter and the sense of togetherness facilitated through group activities are essential to improving health and wellness. Whether that includes playing cultural games, like the *Lahal* bone stick game, or through storytelling, singing, and dancing, these activities were described as deeply meaningful to the participants [46]. These opportunities also allow for language revitalization to occur, where participants can pass on traditional knowledge, a practice that has been used by Indigenous people for generations [11, 47, 48].

By respecting and incorporating these recommendations expressed here (Table 4), future physical activity programs can reflect community strengths and support land-based activities to enhance the health and wellness of all, including those who experience chronic conditions [21, 32].

**Table 4:** Considerations for physical activity service providers based on key findings

Practice Recommendations		
**Clinician (e.g., physiotherapists)**	**Organizations (e.g., CSFS)**	**Regional decision-makers (e.g., Indigenous Services Canada)**
Support walking as a low-cost activity by tailoring recommendations to individual mobility, safety, and local environmental conditions	Offer group- and family-based physical activity events that strengthen kinship, social support, and intergenerational knowledge sharing	Allocate funding for outdoor infrastructure that supports land-based physical activity in rural and remote communities
Design programming that is inclusive of individuals with diverse chronic health conditions through flexibility and accommodations	Implement programming for youth and Elders that reflects their distinct cultural significance within communities	Develop policies that enable sustainable community-led, culturally-focused physical activity programming
Educate patients on the potential use of wearable technologies to support goal-setting, self-monitoring, and reduce sedentary behavior	Co-design physical activity programs with community members to reflect cultural practices, goals, and relationships to land	Add Indigenous voices (e.g., Chiefs, Elders) on environmental and climate considerations to protect land-based practices and activities

### Consideration and limitations

There are some considerations when interpreting these findings. While we aimed to recruit an equal number of participants of all genders, the higher number of women may be influenced by the positionality of the researcher conducting the interviews and the higher prevalence of certain chronic conditions in this gender [8, 13]. Hearing primarily from women may have contributed to the greater emphasis on preferences for women-specific physical activity programming. However, we acknowledge that there may be an equal preference for men’s programming that was not adequately voiced in this study. Therefore, we recommend that there be a balanced focus on developing programming for all genders. In addition, while we coordinated with community leadership to facilitate transportation from participants' homes to the interview locations, we may still have missed hearing from individuals experiencing severe conditions (e.g., fatigue, pain, mobility issues). It is crucial that future programming considers these individuals and provides the necessary accommodations to ensure their participation. In reference to community recruitment, due to the large geography and number of Nations and Bands served by CSFS, we chose to focus on three communities where existing relationships were strongest. These communities also demonstrated a keen interest in continuing collaboration and aided our research endeavors by coordinating meetings, organizing our visits, and providing space to conduct interviews. Finally, as our partnerships with CSFS and communities continue to strengthen, there is an opportunity to involve members from the community more directly in the analytical process. For example, involving Elders and knowledge keepers could provide valuable insights during the data interpretation. Future projects in this collaboration will aim to address all these considerations to enhance the depth and relevance of our findings.

### Future directions

Future research should extend beyond individual-level perspectives to examine community-, organizational-, and systems-level factors that shape physical activity opportunities in rural and remote First Nations contexts. In particular, engaging leadership from community and health service providers, like CSFS, may provide critical insight into how culturally grounded, land-based physical activity initiatives can be implemented and sustained within communities. Emerging technologies, including wearable devices, may offer promising opportunities in sustainable physical activity behaviors in rural and remote contexts; however, further research is needed to address challenges related to data validity, access, and equity [45]. Meaningful community engagement and Indigenous governance over data collection and use will be essential to ensure that these approaches support community priorities [20, 45]. Longitudinal partnerships will be particularly valuable for evaluating the longer-term health impacts of culturally grounded, land-based physical activity initiatives, including their potential influence on chronic disease risk factors and the prevalence and management of chronic health conditions over time.

## Conclusion

This study explored the qualitative physical activity experiences among First Nations people living in rural and remote BC communities. Findings suggest that physical activity is an essential element of the culture of many Carrier and Sekani people. Even before colonization, physical activity was an integral component of their identity and traditional practices. Changes specific to each community are needed to restore that practice to elicit the most beneficial improvements to their health and wellness. These findings align with similar studies exploring the current experience of being active among other Indigenous groups [34, 35, 47]. By centering community voices and priorities in this way, can we then start to address the current gap in health outcomes between First Nations people in BC, which will subsequently answer calls to action that have continued to be made to build an equitable, stronger future for all BC First Nations people [13, 18, 49]. Adhering to culturally sensitive and community-focused approaches can allow us to progress toward health equity through physical activity, which honors the empowerment and strength of First Nations communities [18, 49]. This, in turn, will reduce the disproportionately high rate of chronic conditions faced by First Nations people now and for many generations to come.

## Data Availability

All data supporting the findings of this study are available within the paper. Any further data is protected under community governance and may only be accessed at the discretion of the participating First Nations communities in accordance with OCAP® (Ownership, Control, Access, and Possession) principles.
